# Kinetics of Heterogeneous Background in Stargardt’s Disease over Time

**DOI:** 10.3390/life12030381

**Published:** 2022-03-06

**Authors:** Eduardo Rodríguez-Bocanegra, Marc Biarnés, Míriam Garcia, Lucía Lee Ferraro, Manuel Dominik Fischer, Jordi Monés

**Affiliations:** 1 Barcelona Macula Foundation: Research for Vision, 08022 Barcelona, Spain; mbiarnes@institutmacula.com (M.B.); mgarcia@barcelonamaculafound.org (M.G.); lferraro@institutmacula.com (L.L.F.); jmones@institutmacula.com (J.M.); 2 Centre for Ophthalmology, University Hospital Tübingen, University Eye Hospital, 72076 Tübingen, Germany; dominik.fischer@uni-tuebingen.de; 3 Centre for Ophthalmology, Institute for Ophthalmic Research, University Hospital Tübingen, 72076 Tübingen, Germany; 4 Institut de la Màcula, Hospital Quirón Teknon, 08022 Barcelona, Spain; 5 Oxford Eye Hospital, Oxford University NHS Foundation Trust, Oxford OX3 9DU, UK; 6 Nuffield Laboratory of Ophthalmology, Department of Clinical Neurosciences, University of Oxford, Oxford OX3 9DU, UK

**Keywords:** definitely decreased autofluorescence, fundus autofluorescence, heterogeneous background, foci with reduced autofluorescence, kinetics, Stargardt’s disease

## Abstract

Stargardt’s disease (STGD1) is caused by mutations in the *ABCA4* gene. Different lesions characterised by decreased autofluorescence levels are found in fundus autofluorescence (FAF) from STGD1 patients and could be used as outcome indicators for disease progression. We investigated the fate of foci with reduced autofluorescence (FRA) within the heterogeneous background of STGD1 patients using FAF imaging. Genetically confirmed STGD1 patients presenting heterogeneous background autofluorescence on high-quality FAF images at a minimum of two visits at least 12 months apart were chosen. A grid centred on the fovea was used to define five different zones. Within each zone, five FRA were randomly selected for each eye. The eccentricity of foci was determined at different time points for each patient. Analysis of 175 randomly chosen FRA showed consistent centrifugal displacement over time, most notably in eyes showing areas with definitely decreased autofluorescence. Interestingly, FRA did not leave an area of hypo-autofluorescence on FAF in locations where they were previously located. These findings may help to better understand STGD1 progression, improve FAF interpretation, and shed light on the nature of heterogeneous background.

## 1. Introduction

One of the most common forms of juvenile macular degeneration is Stargardt’s disease (STGD1; OMIM 248200), with an estimated prevalence of 1:10.000 [1]. This autosomal recessive macular dystrophy is caused by mutations in the *ABCA4* gene, and it usually manifests in childhood or adolescence with progressive loss of central vision leading to legal blindness [2,3]. The reason lies in the fact that protein ABCA4 deficiency causes an increased accumulation of lipofuscin components (bisretinoid fluorophores) in the photoreceptor outer segment disc membranes [4]. Then, as a result of outer segment disc separation and phagocytosis by the retinal pigment epithelium (RPE), lipofuscin accumulation increases in the RPE and eventually leads to cell death and macular atrophy [5].

Although STGD1 disease still has no cure, new promising studies involving cell and gene therapies are being developed as potential avenues of treatment [6,7]. However, the best approach to track the progression of the disease and which endpoints to use to assess therapy impact are debated. The Natural History of the Progression of Atrophy Secondary to Stargardt’s Disease (ProgStar) studies were launched in 2013 to help prepare for future clinical trials by using fundus autofluorescence (FAF) to determine disease progression [8]. 

As a non-invasive imaging method, FAF can reveal atrophic lesions and abnormalities in lipofuscin accumulation that were otherwise undetectable with regular colour fundus photography. At least three different lesions characterised by decreased FAF levels can be observed in the fundus of patients with STGD1. These indicate different underlying structural abnormalities, and thus it is important to differentiate them. “Definitely decreased autofluorescence” (DDAF) areas were defined in the ProgStar study as areas with 90–100% darkness in comparison to the optic disc, and are regarded as areas devoid of RPE [8,9]; “questionably decreased autofluorescence” (QDAF) areas were defined in the same study as regions with 50–90% darkness in comparison to the optic disc, and are thought of as a state of transition from normal autofluorescence to DDAF [8,9,10]; and “heterogeneous background” consists of widespread foci of increased or reduced autofluorescence for which no clear anatomic or structural correlate has been established [9].

Therefore, a better understanding of the characteristics and progression of the heterogeneous background is required to gain insights into the phenotypic manifestations of STGD1. The purpose of this study is to investigate the fate of foci with reduced autofluorescence (FRA) within the heterogeneous background of STGD1 patients over time using FAF imaging.

## 2. Materials and Methods

### 2.1. Design

This was a retrospective observational study of patients with STGD1 examined at the Institut de la Màcula (Hospital Quirón Teknon; Barcelona, Spain) between 2010 and 2018. This research is part of the STARK project, “Study of Stargardt’s macular dystrophy: kinetics of disease progression”. The study adhered to the tenets of the Declaration of Helsinki and was approved by the Fundación Quirón Salud Ethics Committee. All patients signed informed consent.

### 2.2. Eligibility Criteria

Patients of any gender aged 18 years or older who visited the Institut de la Màcula with a clinical STGD1 phenotype and genetic confirmation of biallelic mutations in the *ABCA4* gene were included. Eligible patients had to have evidence of heterogeneous background and two ophthalmic examinations with colour fundus photography (CFP) and FAF spaced a minimum of 12 months between them. Patients were excluded if they had other concomitant retinal disorders, previous intraocular therapy/surgery for a retinal disease, or poor imaging quality.

Included patients were then stratified according to the presence or absence of concomitant DDAF areas. In bilateral cases, the study eye was randomly chosen.

### 2.3. Procedures

All patients received a complete ophthalmic examination including medical history, best-corrected visual acuity, anterior segment slit-lamp biomicroscopy examination, and applanation tonometry. Retinal imaging was performed after pupil dilatation with 1% tropicamide and 10% phenylephrine, and included 35° non-stereoscopic CFP (TRC 50DX IA^®^; IMAGEnet, Topcon Corporation, Tokyo, Japan), and infrared, FAF (excitation filter at 488 nm; barrier, >500 nm) and SD-OCT using Spectralis HRA+OCT^®^ (Heidelberg Engineering, Heidelberg, Germany). The SD-OCT examinations were acquired with simultaneous visualization of the fundus with FAF or infrared imaging. The acquisition consisted ≥ of 19 horizontal (245 μm between adjacent B-scans), high-resolution (1536 × 1536 pixels) B-scans covering 20° × 15° centred on the foveal avascular zone (FAZ), with a minimum automatic real-time (averaging) of 9. All SD-OCT exams were performed with the eye-tracker activated from baseline. Fluorescein angiography was performed only if needed according to medical criteria.

### 2.4. Processing of FAF Images

**Figure 1:**
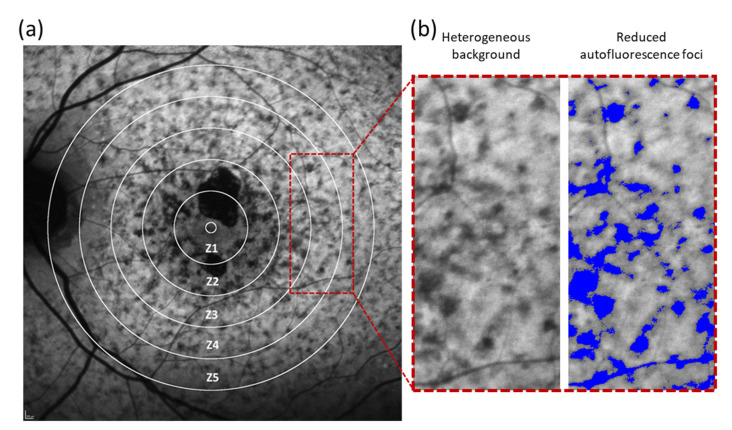
Representative FAF image of a STGD1 patient with heterogeneous background. (**a**) A grid of five concentric white rings with a radius of 0.75, 1.5, 2.25, 3.0 and 3.75 mm centred on the foveola separated five different zones (Z1–Z5, respectively) in the retina. (**b**) Magnified image from FAF image (red rectangle) shows the appearance of heterogeneous background and reduced autofluorescence foci or FRA (in blue).

All images were analysed with Fiji software (ImageJ software, Bethesda, MD, USA). Five concentric rings with a pre-defined, arbitrary radius increasing by 0.75 mm with respect to the previous zone were drawn from the centre of the FAZ on baseline FAF images (Figure 1a). These five rings were required to separate five different regions around the fovea: zone 1 (Z1, 0 to 0.75 mm from the centre of the FAZ), zone 2 (Z2, 0.75 to 1.5 mm), zone 3 (Z3, 1.5 to 2.25 mm), zone 4 (Z4, 2.25 to 3.0 mm) and zone 5 (Z5, 3.0 to 3.75 mm). No account for differences in axial length or magnification between patients was made because the aim was to determine if the progression of FRA differed by distance to the centre of the FAF in a given eye, and not by a precise region in the fundus. Then, all FRA in the heterogeneous background were highlighted in Fiji (Figure 1b).

### 2.5. Measuring the Progression of FRA over Time

The centres of five FRA in each zone were randomly chosen, regardless of their location, size, or shape. The centre of the FAZ was taken as a reference to determine the spatial displacement of the FRA over time. The eccentricity of the FRA was measured at baseline and at the last follow-up visit. The median rate of displacement of FRA was determined (in µm/year) for each zone as the difference in the distance from each FRA to the centre of the FAZ at the last visit subtracted from the distance between the same focus at baseline and divided by the time between visits in years. Serial analysis of fundus images was performed by aligning consecutive FAF images to create a movie file using Fiji software.

### 2.6. Correlation of FRA with Structural SD-OCT

To understand the structural abnormalities potentially linked to FRA, SD-OCT guided by FAF imaging of FRA were assessed at baseline and follow-up. The region of interest in which an FRA was visible on FAF was evaluated with SD-OCT. In follow-up visits, the same areas where FRA were seen at baseline were re-valuated with SD-OCT to study changes associated with the apparent movement of the FRA.

### 2.7. Statistical Analysis

The analyses included descriptive statistics for demographic data using medians (interquartile range, IQR) for quantitative and n (percentage) for categorical variables. The correlation between the distance from the FAZ to the FRA in the heterogeneous background was determined using Spearman’s correlation coefficient.

To account for the intra-zone correlation in each eye with heterogeneous background (up to five FRA in each zone, from Z1 to Z5), generalised estimating equations (GEE) models using an exchangeable correlation matrix were used to determine if the apparent FRA movement, as measured in µm/year, differed between groups, defined by the presence or absence of DDAF.

Analyses were conducted using Excel (Microsoft Office) and Stata IC version 15.1 (StataCorp; College Station, TX, USA). A two-tailed *p*-value ≤ 0.05 was considered statistically significant. No adjustment for multiple comparisons was made.

## 3. Results

### 3.1. Patients

The database of Institut de la Màcula contained 53 cases with a clinical phenotype of STGD1. Twenty-four of them had confirmed mutations in the *ABCA4* gene. For 11 of these STGD1 genetically confirmed patients, longitudinal FAF data with a minimum of 12 months of follow-up with good imaging quality were available, but four of these patients did not show heterogeneous backgrounds and were therefore also excluded. Finally, seven patients met all eligibility criteria. The age of the patients at baseline ranged from 18 to 47 years old with a median age of 35 years (IQR = 11). The median length of follow-up was 13 months (IQR = 6.5). The general demographic and baseline information of these patients is summarized in Table 1.

### 3.2. Movement of FRA in Patients with Heterogeneous Background

Studies have demonstrated that some retinal lesions with characteristic fluorescence patterns appear to move across the fundus in STGD1 patients as the disease progresses. This is the case for the intensely fluorescence foci (flecks) that generally emerge in relatively regular patterns along the central-to-peripheral axis of the retina, and may progress outward in a centrifugal pattern from the central macula [11,12]. Here, we hypothesized that the FRA observed in STGD1 patients show a similar apparent movement with disease progression.

FRA within the heterogeneous background exhibited a median movement of 4.5 µm/year (IQR = 40). However, we found that this movement was different depending on the eccentricity at baseline (Table 2). Foci with slower displacement were found in Z1 and Z2, in which the median progression towards the periphery was 2.8 µm/year (IQR = 3.5) and 3 µm/year (IQR = 104), respectively. In Z3, the velocity increased to 17 µm/year (IQR = 90) but slowed down again to 12 µm/year (IQR = 48.8) and 6 µm/year (IQR = 17.8) when the foci reached the zones furthest from the FAZ, in Z4 and Z5, respectively.

### 3.3. Centrifugal Movement Pattern of FRA

We also found that the observed displacement of FRA was always directed away from the FAZ, i.e., in a centrifugal direction. The distance between the FAZ and the FRA was always greater at the last visit compared to the distance at baseline. Therefore, when the distance from baseline was subtracted from the distance at the final visit, the results were always positive, as shown in Table 2 with the symbol “+”. These displacement rates for each eye represented the average of five measurements in each zone.

**Figure 2:**
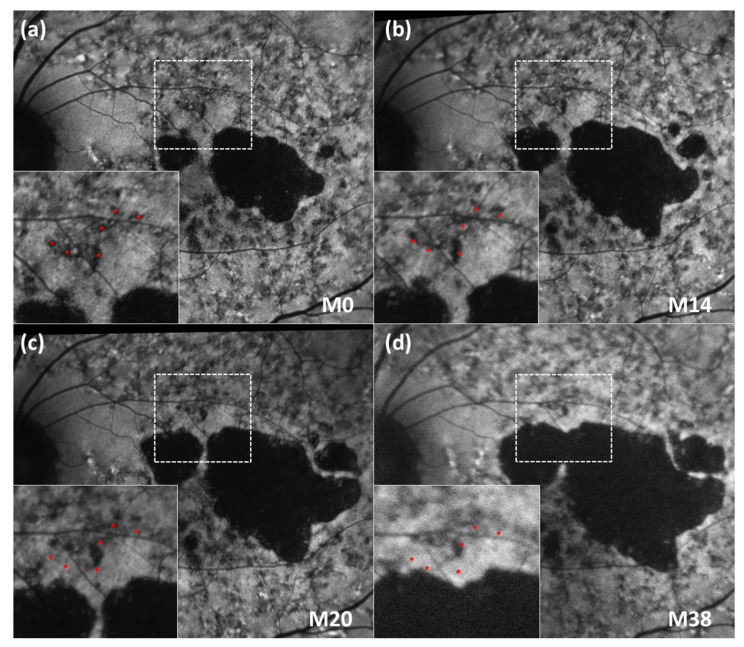
Sequential FAF imaging of an STGD1 patient at different time points. (**a**–**d**) The vessel and highlighted FRA at baseline (in red) are in the same position in every image of the sequence in order to appreciate the movement of the FRA in successive follow-up visits (M0, M14, M20 and M38). FAF: fundus autofluorescence; M: Month; STGD1: Stargardt’s disease.

Examples of the centrifugal movement pattern of FRA in sequential FAF images are shown in Figure 2 and Supplementary Video S1. In the expanded images from Figure 2, several FRA were highlighted around a main blood vessel as a reference. The distance between the vessel and the red asterisks (which indicate the location of the FRA at baseline) was the same in all sequential images. Interestingly, the FRA did not leave behind an area of hypo-autofluorescence on FAF when displaced to more eccentric positions over time (Supplementary Video S1).

### 3.4. Influence of DDAF Areas on Displacement of FRA

It has been estimated that in STGD1, 50% of eyes with no DDAF area at the initial visit will develop such a lesion within 5 years [13]. Thus, we analysed whether the presence and progression of DDAF areas had an impact on the movement of the FRA.

Two groups of patients were defined by the presence or absence of DDAF at the first visit. A comparative analysis of the velocity of movement between the groups revealed that FRA moved faster in patients with presence of DDAF (43.29 µm/year, IQR = 73.5) than in those without (2 µm/year, IQR = 2; *p* = 0.002).

**Figure 3:**
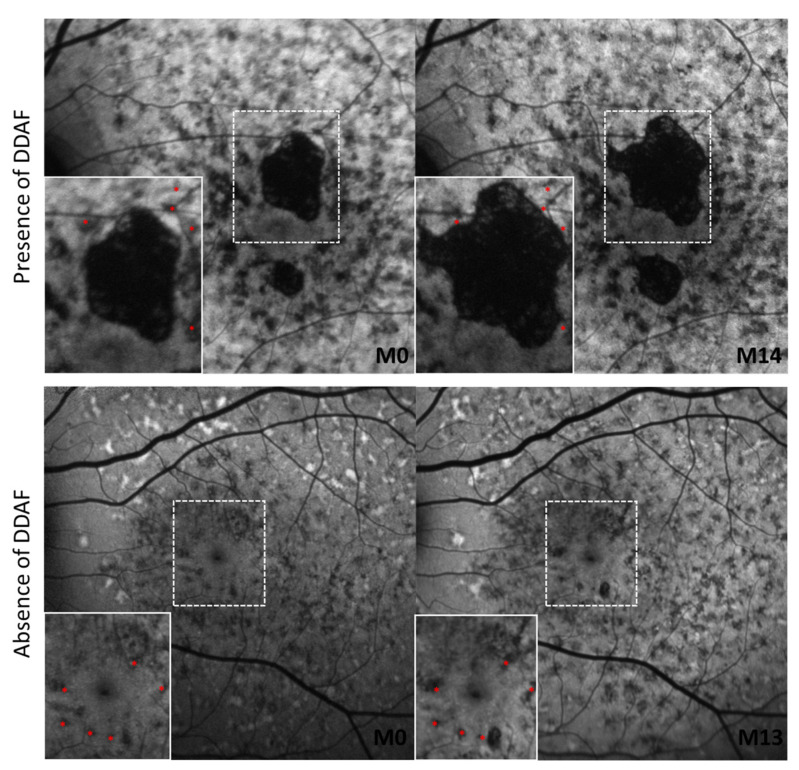
Comparison between kinetics of FRA according to the presence or absence of DDAF lesions. Highlighted FRA at baseline (in red) are always in the same position in order to appreciate the movement of the FRA between two time points. DDAF: definitely decreased fundus autofluorescence; FRA: foci of reduced autofluorescence; M: Month.

We also found that the presence of DDAF influenced the movement of FRA regardless of the zone in which it was located (Table 3). In patients with DDAF areas, the mean FRA displacement rate was 27 µm/year in Z1, 77 µm/year in Z2, 67 µm/year in Z3, 35 µm/year in Z4, and 30 µm/year in Z5. On the other hand, in patients without DDAF, the mean rate displacement ranged from 1 to 3 µm/year in all areas (Figure 3).

### 3.5. Qualitative Analysis of SD-OCT Data at FRA

To elucidate the nature of FRA, we compared SD-OCT imaging information from areas with reduced autofluorescence to healthy or immediately adjacent areas in our patients.

**Figure 4:**
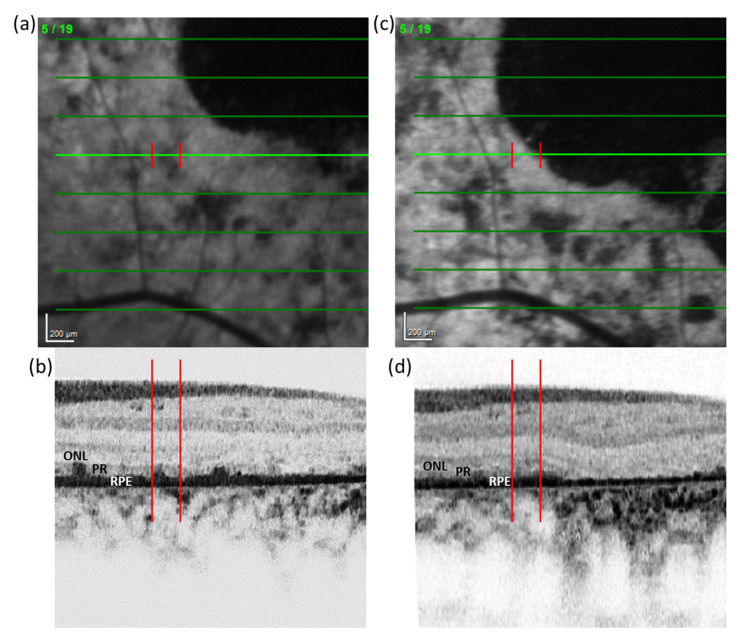
Simultaneous visualization of an FRA in FAF and SD-OCT imaging. (**a**) Reduced autofluorescence focus under the bright green line delimited by two red lines that project on the (**b**) SD-OCT. (**c**) Follow-up of FAF image 30 months later. (**d**) Red lines delimit the same location as in (a-b) from FAF to the corresponding SD-OCT location. FAF: Fundus autofluorescence. ONL: Outer nuclear layer. PR: photoreceptors inner and outer segments. RPE: retinal pigment epithelium. SD-OCT: Spectral-domain optical coherence tomography.

The comparison between the SD-OCT signal in FRA and healthy areas showed that signal attributed to the RPE monolayer was altered, but generally not absent, in zones with FRA. In 57% of cases in which DDAF areas were present in the retina, SD-OCT revealed an apparent increase in the RPE thickness after displacement of the reduced autofluorescence focus to a new location (Figure 4).

In addition, to try to identify the layer(s) potentially involved in originating the FRA, a qualitative comparison between areas with FRA and immediately adjacent areas to them was performed. However, no clear pattern was observed, and therefore the anatomic origin of the FRA signal could not be unequivocally identified.

## 4. Discussion

The lesions we describe herein meet the definition of foci with reduced autofluorescence (FRA) in terms of FAF intensity and topographic distribution within the heterogeneous background, as defined in the ProgStar study [9]. In previously published reports, it has been shown that heterogenous background has a strong influence on the progression of the RPE atrophy or DDAF and is associated with a more rapid functional deterioration [10,14]. In light of this, it is important to deepen the understanding of the heterogeneous background and the progression that helps to define it.

Therefore, in this retrospective study, we investigated the spatial displacement of FRA within the heterogeneous background of STGD1 patients using FAF imaging over time. Our data showed that FRA appear to move over time in a centrifugal fashion as the disease progresses, without leaving behind a low autofluorescence signal. In addition, the movement of these foci was not the same in all areas of the retina we measured. Particularly in areas at 2.25–3 mm from the FAZ (Zones 3 and 4), the movement of FRA was faster than in the other areas. Interestingly, we also found that the movement of FRA was more evident when DDAF areas were present.

It has been previously described that hyperautofluorescent flecks show centrifugal movement in the natural course of the disease [11,12,15,16]. These flecks are an early hallmark of STGD1 which progress by increasing and then decreasing in intensity on FAF imaging over time, leaving residual atrophy [15]. However, our findings describe a new phenomenon in which centrifugal movement occurs in FRA within the heterogeneous background of STGD1 patients. Although FRA could be considered as incipient areas of RPE atrophy due to their lower autofluorescence in comparison to surrounding regions, we showed that in contrast to hyperautofluorescent flecks, FRA did not leave an area of hypoautofluorescence on FAF in locations where they were previously located. It is due to this phenomenon that a potential call for re-appraisal of FAF interpretation could be necessary.

It has been reported that flecks exhibit hyper-reflective, convex, pyramidal-shaped, or ill-defined deposits on SD-OCT, which interfere with the photoreceptor ellipsoid zone and extend into the outer nuclear layer with a remaining connection to the RPE, which eventually undergoes atrophy [17,18]. However, the source of FRA within the heterogeneous background clearly differs from flecks on SD-OCT. Müller et al. described the anatomic and functional changes of different areas of the retina including the heterogeneous background [19]. They revealed a series of FAF patterns in STGD1 that were associated with functional impairment and structural changes on SD-OCT imaging. However, we found that in approximately 60% of the cases where DDAF was present, there was an apparent increase in the thickness of the RPE signal on SD-OCT after displacement of the FRA to a new location. Nevertheless, a larger study should be performed in order to validate these observations.

In a subgroup analysis between patients with or without DDAF, we observed that FRA displacement was more marked in eyes with DDAF. This relationship is in line with previous reports suggesting that the heterogeneous background developed more rapidly with the growth of the DDAF lesion [14].

**Figure 5:**
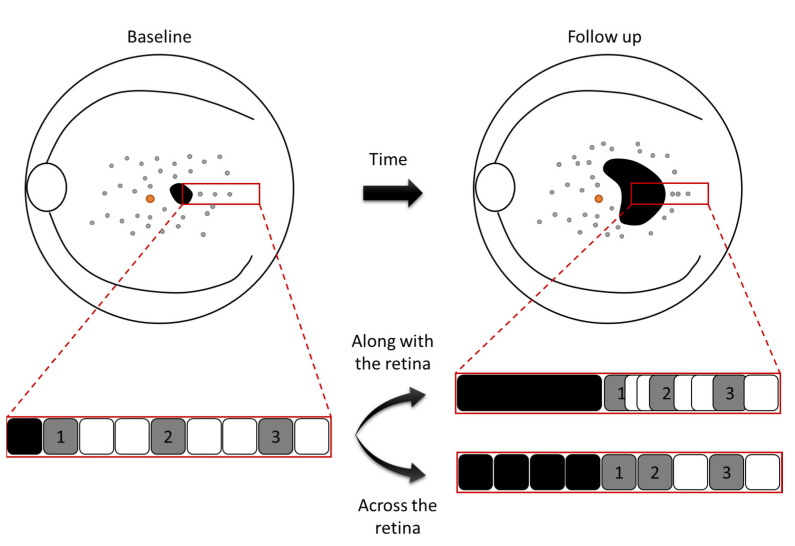
Graphic representation of two hypothetical scenarios to explain movement of FRA within the heterogeneous background. The movement could potentially occur either along with or across the retinal tissue over time. The squares in the red rectangles represent retinal tissue (black: definitely decreased autofluorescence; grey: foci of reduced autofluorescence).

Unfortunately, the mechanism of FRA displacement remains unknown. From our observations, the movement could potentially occur either along with or across the retinal tissue (Figure 5). If the movement occurred along with the tissue, this would mean that the overall tissue would contract away from the location of DDAF. This hypothesis would be supported by our observations in which the movement of FRA is faster when DDAF areas are present. Additionally, the degree of contraction would be greater in areas surrounding the FAZ (originating regions of atrophy) resulting in a greater displacement of lesions close to these areas, but smaller in more distal zones. On the other hand, if the movement occurred across the tissue, this would mean that the FRA would migrate through the retina and be potential indicators where atrophy will “pass” through. Nevertheless, these are just hypotheses. The relationship between FRA and photoreceptor and/or RPE loss is unclear and beyond the phenomenon discussed herein. However, it is the topic of ongoing work, in which we are trying to correlate in a masked fashion the presence of FRA with abnormalities in different retinal layers, as seen on SD-OCT in several regions of interest.

One potential limitation of this study is the number of patients. In order to validate these observations, a larger study should be performed with a larger number of cases. However, it must be noted that our unit of analysis was the total number of FRA, which was much larger (175) than the number of eyes (7). In addition, other imaging methods such as near-infrared autofluorescence (PMID 26230768) or fluorescence lifetime imaging ophthalmoscopy or FLIO (PMID 28673870) may be useful here, but unfortunately, they were not available in the current study. In this sense, the inclusion of FLIO may also reveal interesting clues regarding the nature of FRA.

In conclusion, FRA in the heterogeneous background showed a centrifugal displacement over time, which was increased in the presence of DDAF. The results of this study provide more information to help us understand disease progression and FAF interpretation, and may contribute to disentangling the formation of these lesions in STGD1 patients.

## Figures and Tables

**Table 1:** Baseline characteristics of the patients included in the study. BCVA: best-corrected visual acuity (number of letters on the Early Treatment Diabetic Retinopathy Study chart with the Snellen Equivalent in brackets); F: female; M: male; *: stop codon.

Patient #	Age (Years)	Sex	Duration of Symptoms (Years)	ABCA4 Mutations	BCVA	Follow-Up (Months)
1	23	F	18	p.Arg212Cys; c.4253 + 5G > A	46 (20/125)	14
2	47	F	2	p.Trp700Leu; p.Leu1850Pro	57 (20/80)	30
3	34	M	1	p.Pro1486Leu; p.Gly1977Ser	90 (20/15)	12
4	42	F	27	p.Arg408X; p.Arg1129Leu	35 (20/200)	14
5	18	F	3	p.Trp439 *; c.5714 + 5 G > A	85 (20/20)	13
6	36	M	2	p.Trp700Leu; p.Leu1850Pro	91 (20/15)	12
7	45	F	25	p.Arg212Cys; p.Gly1961Glu	55 (20/80)	12

**Table 2:** Summary of the median rate of FRA movement in Zones 1 to 5. DDAF: definitely decreased autofluorescence; IQR: interquartile range; *: only DDAF lesion in the Zone; †: no FRA in the Zone.

Patient #	DDAF	Median Rate of FRA Movement (µm/year)				
		Zone 1	Zone 2	Zone 3	Zone 4	Zone 5
1	Present	*	128	101	50	13
2	Present	*	*	84	52	39
3	Present	5	83	17	14	1
4	Present	*	*	*	10	9
5	Absent	1	1	1	1	2
6	Absent	1	2	4	2	3
7	Absent	3	3	†	†	†
Median (IQR)		+2.8 (3.5)	+3 (104)	+17 (90)	+12 (48.8)	+6 (17.8)

**Table 3:** Comparison of FRA movement rate (in µm/year) between eyes with and without DDAF by zone. Distances are taken from the centre of the foveal avascular zone. CI: confidence interval; DDAF: definitely decreased fundus autofluorescence.

Zone	DDAF Present (µm/year)	DDAF Absent (µm/year)	Present–Absent (95% CI) (µm/year)	*p*-Value
1	27	2	+25 (−1 to 52)	0.05
2	77	2	+75 (38 to 112)	< 0.001
3	67	1	+66 (6 to 105)	0.001
4	35	2	+34 (−8 to 75)	0.11
5	30	3	+26 (−39 to 73)	0.31

## Data Availability

Not applicable.

## Supplementary Materials

The following supporting information can be downloaded at: https://www.mdpi.com/article/10.3390/life12030381/s1, Video S1: Sequential FAF imaging of a STGD1 patient showing the movement of FRA (in blue) in heterogeneous background over time. FRA are labelled in red to indicate their position at baseline.
