# Supplementary material for: Kinetics of Heterogeneous Background in Stargardt’s Disease over Time
Source: Life (Basel). 2022 Mar 6;12(3):381. doi: 10.3390/life12030381 (PMC8953836; doi:10.3390/life12030381)
Supplement: Supplementary file 1 [file life-12-00381-s001.zip › life-1595580-supplementary.pdf]

life

MDPI

Supplementary material of Kinetics of Heterogeneous Background in Stargardt's Disease Over Time

Video S1: Sequential FAF imaging of a STGD1 patient showing the movement of FRA (in blue) in heterogeneous background over time. FRA are labelled in red to indicate their position at baseline.
